# Supplementary material for: Creation of New Oregano Genotypes with Different Terpene Chemotypes via Inter- and Intraspecific Hybridization
Source: Int J Mol Sci. 2023 Apr 15;24(8):7320. doi: 10.3390/ijms24087320 (PMC10138667; doi:10.3390/ijms24087320)
Supplement: Supplementary file 1 [file ijms-24-07320-s001.zip › Supplemental Figures.pdf]

Supplementary Figures

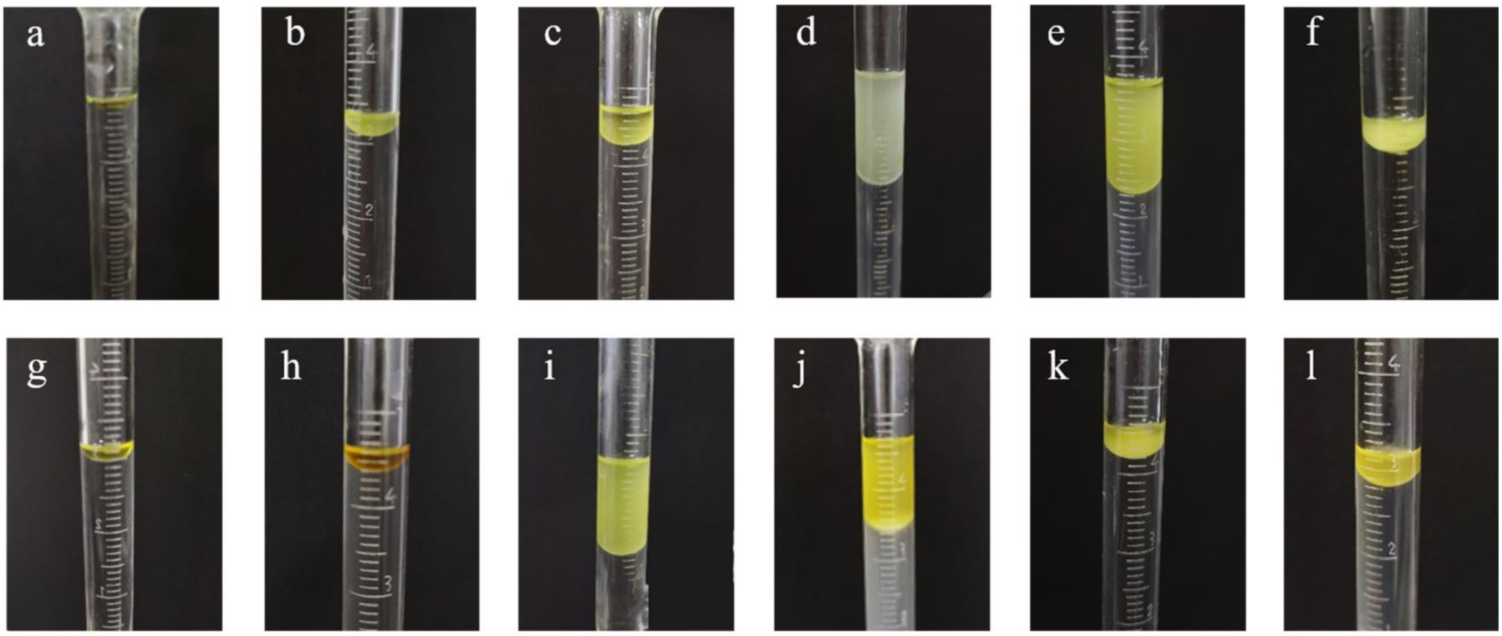

Fig. S1. Images of the twelve essential oil chromatograms of different oregano genotypes. (a) *O. vulgare* (Ov). (b) *O. vulgare* 'Creticum' (Ovc). (c) *O. vulgare* 'Golden Shine' (Ovgs). (d) *O.* × *majorana* 'Hippokrates' (Omh). (e) *O. vulgare* 'Hot&Spicy' (Ovhs). (f) *O. vulgare* 'Aurea' (Ova). (g) *O. vulgare* 'Aureum Gold' (Ovag). (h) *O. laevigatum* 'Rosenkuppel' (Olr). (i) *O. vulgare* 'Samothrakb' (Ovs). (j) *O. vulgare* subsp. *hirtum* (Ovh). (k) *O. vulgare* 'Thumble's variety' (Ovt). (l) *O. vulgare* 'Varona' (Ovv).

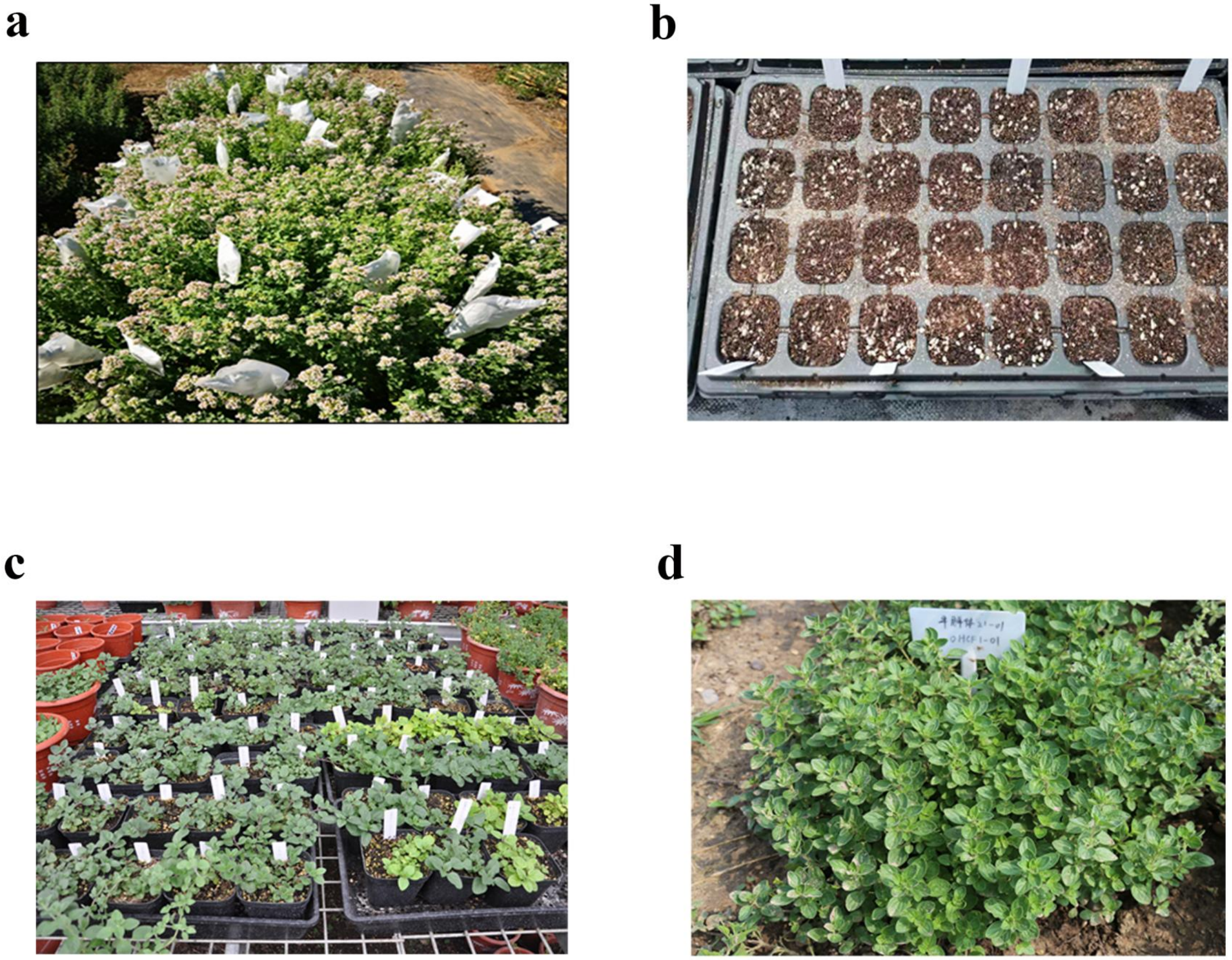

Fig. S2. Process of the generation of six hybrid combinations of oregano and their F_1 lines. (a) Oregano hybridization process. (b) Sowing process. (c) Seedling stage. (d) The hybrid seedlings were transplanted to the resource garden.

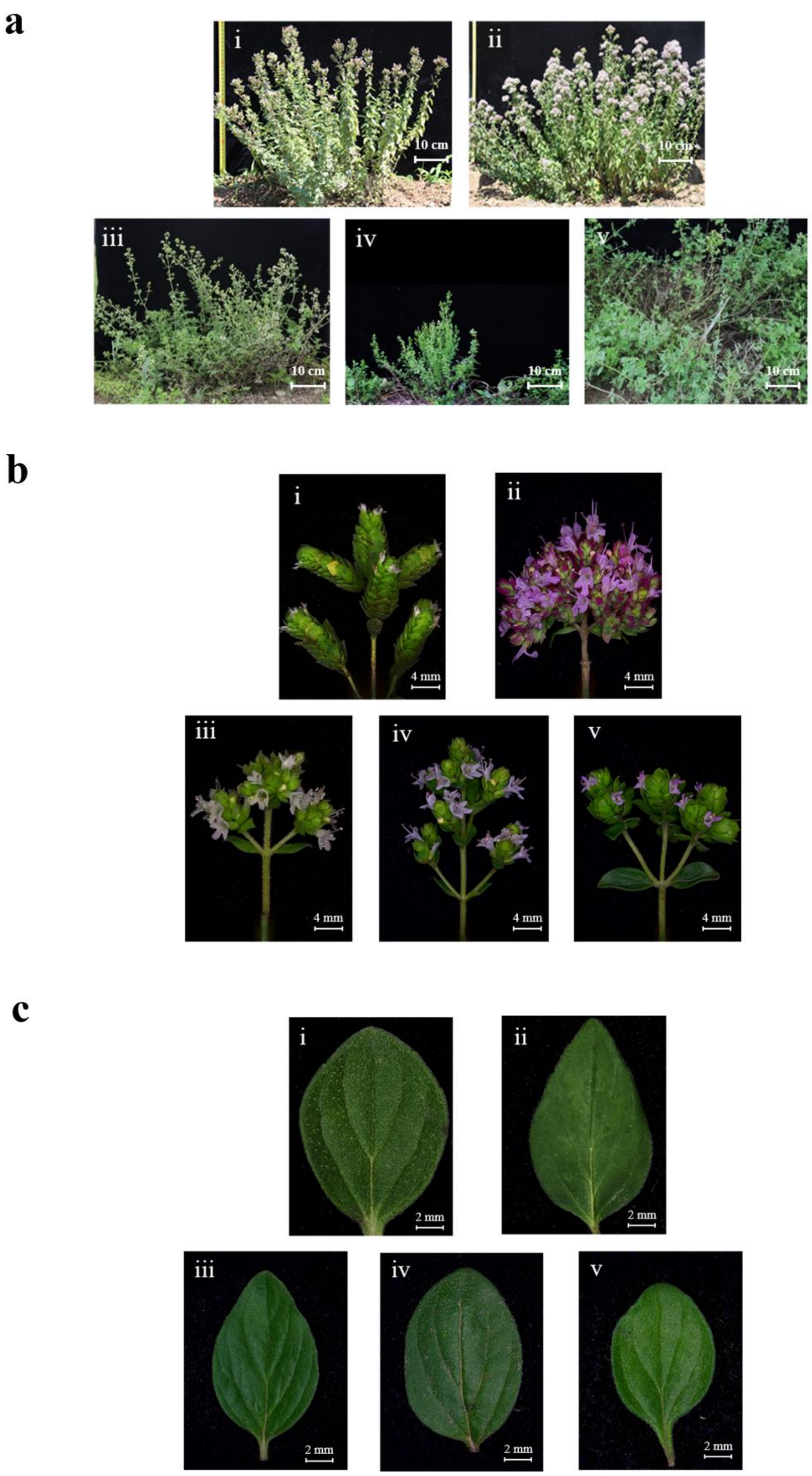

Fig. S3. Plant type, leaf and flower type of *O.* × *majorana* 'Hippokrates' (Omh) × *O. vulgare* 'Creticum' (Ovc) progeny. (a) Plant type. (b) Flower type. (c) Leaf type. i Female parent Omh; ii Male parent Ovc; iii F_1-2; iv F_1-5; v F_1-11.
